# Chemo-Mechanical Approach to Improve Repair Bond Strength of Denture Teeth

**DOI:** 10.1155/2020/8870361

**Published:** 2020-11-04

**Authors:** Zahra A. AlZaher, Danah F. Almaskin, Masoumah S. Qaw, Tahani H. Abu Showmi, Reem Abualsaud, Sultan Akhtar, Mohammed M. Gad

**Affiliations:** ^1^College of Dentistry, Imam Abdulrahman Bin Faisal University, P. O. Box 1982, Dammam 31441, Saudi Arabia; ^2^Department of Substitutive Dental Sciences, College of Dentistry, Imam Abdulrahman Bin Faisal University, P. O. Box 1982, Dammam 31441, Saudi Arabia; ^3^Department of Biophysics, Institute for Research and Medical Consultations, Imam Abdulrahman Bin Faisal University, P. O. Box 1982, Dammam 31441, Saudi Arabia

## Abstract

**Background:**

Detachment of acrylic teeth from denture base material is a common complication in dentistry which accounts for 26–30% of repair cases. This study aimed to evaluate the effect of alumina-blasting, silane coupling agent, and thermal cycling on the shear bond strength of repaired teeth to denture base.

**Materials and Methods:**

Specimens (140) of repaired teeth to denture bases were fabricated and divided into 14 groups: 7 groups before thermal cycling and 7 groups after thermal cycling (*n* = 10). The groups were divided according to surface treatment into no treatment (control), treatment of the base (B), the tooth (T), or both (BT). Each group was further subdivided according to the surface treatment method into alumina-blasting or alumina-blasting and silane coupling agent. After treatment, acrylic discs and teeth were fixed in a jig, and the repair procedure was done. Half the specimens were thermally cycled. Shear bond strength was measured using a universal testing machine. ANOVA and Tukey HSD tests were performed at *α* = 0.05.

**Results:**

Surface treatment significantly improved the bond strength compared to the control group (*P* < 0.001). Comparing surface treatments, alumina-blasting with silane coupling agent treatment resulted in significantly higher strength compared to alumina-blasting alone (*P* < 0.001). The BT group treated with alumina-blasting and silane coupling agent showed the highest significant shear bond strength (23.91 ± 0.96 MPa) (*P* < 0.001). Significant drop in strength value was observed in all groups after thermal cycling (*P* < 0.004) except the BT group treated with alumina-blasting (*P*=0.096).

**Conclusion:**

Surface treatment using alumina-blasting with silane coupling agent for denture base and tooth increased repair strength.

## 1. Introduction

The commonest used material to fabricate denture base resins and artificial acrylic teeth for complete and partial dentures is poly methyl methacrylate (PMMA) [1, 2]. Acrylic teeth made of PMMA are preferred over porcelain teeth due to several advantages: low cost, wide availability, ease of manipulation, chemical bond to denture base, and esthetic acceptability even in thin sections [3]. Although PMMA has all the mentioned advantages, it has few drawbacks including dimensional changes, porosities, poor mechanical resistance, and possible allergic reaction in some patients [1, 2, 4]. Acrylic resin artificial teeth should be well bonded to the denture base to promote the prosthesis success rate, especially with increased chewing forces in implant-retained and -supported overdentures [5]. Debonding or detachment of these artificial teeth from the denture base is a common complication resulting in 26–30% of repair cases [6]. It is recommended to remove any residual wax from the teeth ridge-lap surface before processing, as it can lead to teeth debonding [7]. Teeth detachment or failure consumes the dentist's time and effort as well as adds cost for the patient [6].

The standard technique to rebond the artificial acrylic teeth to the denture base is by using autopolymerizing acrylic resin [8]. Surface treatment of either the artificial teeth or the denture base is needed for shear bond strength (SBS) improvement and to overcome teeth debonding and separation from the denture base [9]. All previous studies tried to improve the strength using different surface treatments of the acrylic teeth only [6, 9–11]. Several authors suggested treating the tooth surface before processing with chemicals like methyl acetate-based bonding agent along with diatoric recess to improve the SBS [9]. Mechanical treatment alone like roughening the surface by sandblasting or combining mechanical and chemical surface treatments such as sandblasting followed by bonding agent (monomer-based) application or tribochemical coating improved the bond strength [10]. Furthermore, mechanical roughening of the ridge-lap surface of the tooth before processing using a round bur improved the SBS of artificial acrylic teeth to the denture base [11].

One of the known adhesive materials is silane coupling agent (SCA). It is capable of creating chemical bonds with repair resins with the help of unconverted C═C double bonds [12–14]. Qaw et al. [15] concluded that the bond strength of repaired acrylic denture was enhanced by the application of SCA on a mechanically treated surface. A study by Lang et al.[10] found that tribochemical silica coating and salinization using SCA on the ridge-lap side of the teeth before denture processing resulted in significant improvement of bond strength; hence, both techniques were recommended to enhance the bond strength.

Even with the researches done previously, debonding of repaired teeth was reported as adhesive failure in most of the cases [9]. The current study aimed to assess the effect of different surface treatments of acrylic tooth, base, or both and thermal cycling on the SBS of the repaired artificial acrylic tooth to PMMA denture base resin. The null hypothesis was that SBS between the tooth and the base would not change after surface treatments or thermal cycling.

## 2. Materials and Methods

Based on previous studies [8, 9], sample size calculation revealed that a total of 140 specimens (70 before and 70 after thermal cycling) were suitable to conduct the current study. The acrylic specimens were randomly divided into 14 groups: 7 groups before thermal cycling and 7 groups after thermal cycling (*n* = 10) (Table 1). Specimens were subdivided according to the side of surface treatment into control group (no treatment), base group (B), tooth group (T), and base + tooth group (BT). Each treated group was further subdivided into two groups according to the type of surface treatment: airborne alumina particle abrasion which was denoted as AB for alumina-blasting and surface treatment with silane coupling agent which was denoted as SCA (Table 1).

**Figure 1:**
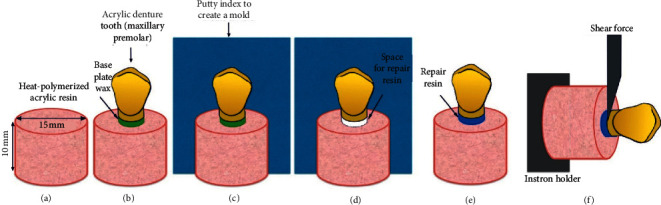
Diagrams representing specimen preparation and testing: (a) acrylic resin disc; (b) acrylic tooth waxed on resin disc; (c) repair mold preparation; (d) repair procedure and assembly of heat-polymerized acrylic disc and tooth; (e) repaired specimen; (f) shear bond strength test illustration.

Heat-polymerized acrylic resin specimens were fabricated to represent the denture bases (Major.Base.20; Major Prodotti Dentari Spa, Moncalieri, Italy) (Figure 1). First, a metal disc (15 mm × 10 mm) was used to create silicon molds that were used to fabricate 140 wax specimens (Cavex Set Up wax; Cavex, Haarlem, the Netherlands). Second, investment of the wax specimens was done in a conventional manner using dental stone (Fujirock EP; GC Europe, Leuven, Belgium) inside a metal flask (61B Two Flask Compress; Handler Manufacturing, Westfield, New Jersey). After that, the wax was eliminated using a wax elimination machine for 10 minutes to create mold spaces. A separating medium (Iso Major; Major Prodotti Dentari Spa, Moncalieri, Italy) was applied to all stone surfaces and left to dry. Then, heat-polymerized acrylic resin was prepared and mixed as instructed by the manufacturer and packed at dough stage under pressure (3500 psi) using a pneumatic press until excess material was seen, and then the flask was left aside in a flask clamp under tight pressure for 30 minutes. For polymerization, the flasks were immersed into room temperature water within the thermal curing unit (KaVo Elektrotechnisches Werk GmbH, Biberach, Germany) and processed at 74°C for 8 hours followed by 100°C for 1 hour. Once polymerized, flasks were recovered, allowed to bench-cool for 30 minutes, and then opened. Acrylic specimens were retrieved and finished using a straight handpiece with the ISO 040 carbide acrylic bur (Zhangjiagang Saimeng Tools Co. Ltd., Jiangsu, China) and then ground flat with 600-grit silicon carbide paper to eliminate any irregularity. Finally, all specimens were kept in water at 37°C for 2 days [16].

After the heat-polymerized acrylic resin specimens were fabricated, one maxillary first premolar (Yamahachi Dental MFG., CO., Aichi, Japan) was fixed to the prepared specimens using wax (2 mm in thickness) [17] to create space representing the recess prepared during denture repair into which repair resin would be packed later (Figure 1). A new silicon mold was prepared for the waxed-up specimen and used as a holder for the acrylic disc and tooth to standardize all specimens' repairs (Figure 1).

After mold preparation, surface treatments were done for either the base, the tooth ridge-lap surface, or both (Table 1). The assigned surfaces for AB were mechanically treated by aluminum oxide particles (50 *μ*m) (Korox50, Bego Bremer Goldschlagerei, Wilh. Herbst GmbH & Co. KG, Bremen, Germany) using a sandblasting machine (Wassermann Dental-machine, CEMAT-NT3, GMBH, Hamburg, Germany) for 10 seconds at 2.5 bar pressure and 10 mm distance between the source (nozzle) and the treated surface [15]. After that, loose debris was removed using an air-water spray for 10 seconds, followed by drying using compressed air for 3 seconds. The alumina-blasting procedure was done for all the specimens by one trained person. After AB surface treatment, specimens assigned to receive SCA treatment were treated as follows: a micro brush was used to apply a thin single layer of SCA (Shanghai Richem International Co., Ltd. Shanghai, China) onto the AB-treated surface and then left aside for 20 minutes to allow for acetone evaporation.

After surface treatment and within the mold, each acrylic disc and tooth was assembled (Figure 1). Following the manufacturer's instructions, autopolymerized acrylic resin (Major repair; Major Prodotti Dentari Spa, Moncalieri, Italy) was mixed and packed in the repair area. Repair material was polymerized under 2 bar pressure at 37°C for 10 minutes using a polymerization pressure vessel. After complete polymerization, repaired specimens were finished using a straight handpiece and carbide acrylic bur (Figure 1). After that, specimens were kept in distilled water at 37°C for 48 hours [16]. Half the specimens were thermally cycled using a thermal cycling machine (Thermocycler THE-1100-SD Mechatronik GmbH, Feldkirchen-Westerham. Germany), where they were subjected to 5,000 cycles [16, 18, 19] between 5 and 55°C [19] with 1-minute dwell time.

The SBS (MPa) of the repaired specimens was tested using the universal testing machine (Instron 8871; Instron Co., Norwood, Massachusetts, USA). Shear force using 5 kN load cell [19] was applied using a knife-edge-shaped tip positioned parallel to the bonded surface at a crosshead speed of 1 mm/min (Figure 1) [19]. The maximum force needed to break the repaired specimens was identified in newton (N), and the SBS was calculated using the formula *R*=*F*/*A* where “*R*” is the shear bond strength (MPa), “*F*” is the force needed to separate the specimen, and “*A*” is the area of the interface (mm^2^), *A*=*πr*^2^, where *r* is the radius of the tooth base.

Surface morphology changes after surface treatment and after fracture were examined using a scanning electron microscope (SEM) (Inspect S50, FEI, Brno, Czech Republic). Ten specimens from each main group (control, B, T, and BT) were examined using the SEM. Fractured specimens were gold-coated using a sputter coating machine (Q150R ES, Quorum, East Sussex, UK) to overcome the nonconductive nature of the material. Nature of failure was determined by naked eye visual examination in addition to SEM micrographs taken at different magnifications. The nature of failure was considered adhesive when the fracture happened at the interface between the repair resin and denture base or acrylic tooth and/or if 25% or less of the repair resin was observed on the base material or the tooth. Cohesive failure was considered when the fracture occurred within the base, the tooth, within the repair resin, or when the repair material was observed on the tooth or base surfaces by more than 75%. Mixed failure was considered when 25–75% of repair resin was found at the interface [15]. Finally, the topography of the fractured surfaces was analyzed for mode of fracture as brittle or ductile fracture.

Statistical package of social sciences (SPSS V. 21, IBM software, Chicago, USA) was used for data entry and analysis. Data were presented as means and standard deviations and were found to be normally distributed after evaluation using the Kolmogorov–Smirnov test. Analysis of variance (ANOVA) was used to identify the differences among different groups before and after thermal cycling. Furthermore, SBS before and after thermal cycling was analyzed independently considering thermal effect on different treatment groups. At first, ANOVA was used to analyze treatment groups and the control group all together. After that, the Tukey Honestly Significant Difference (HSD) multiple comparisons test was used to make the post hoc comparisons and identify significant differences at *α* = 0.05. For the nature of failure analysis, a Mann–Whitney test was performed to detect any significant difference before and after thermal cycling.

## 3. Results

One-way ANOVA results showed significant differences between groups before (*F* = 245.11, *P* < 0.001) and after (*F* = 447.53, *P* < 0.001) thermal cycling.  Table 2 represents the mean SBS values and standard deviations of the study groups and the significance between them. As shown in Table 2 and before thermal cycling, all the surface-treated groups had significantly higher SBS compared to the control group (*P* < 0.001). Comparing the surface of treatment, there was no significant difference of SBS between B-AB and T-AB (*P*=0.058); however, treatment of both base and tooth surfaces with AB resulted in significantly higher SBS value of BT-AB compared to single surface counterparts (*P* < 0.001). All chemomechanically (alumina-blasting and SCA) treated groups were significantly different from each other (*P* < 0.001) with the BT-AB + SCA group showing the highest SBS value (23.91 ± 0.96 MPa). Looking at the type of treatment, the results showed that the combined treatment of the surfaces with alumina-blasting and SCA resulted in significantly better SBS than alumina-blasting alone for the B and BT groups only (*P* < 0.001). Out of all nonthermal cycled test groups, T-AB showed the lowest SBS value (15.81 ± 0.60 MPa).

After thermal cycling, the results showed that all treated groups had significantly higher SBS compared to the control group (*P* < 0.001). Looking at the effect of the treatment on the surface, there were significant differences between all groups of treated surfaces per type of treatment, between B-AB, T-AB, and BT-AB (*P* < 0.001 for all except between B-AB and T-AB, *P*=0.046) and between B-AB + SCA, T-AB + SCA, and BT-AB + SCA (*P* < 0.001). The same trend was seen before and after thermal cycling where BT showed the highest SBS value followed by B groups and then T groups. When comparing the type of treatment, there was a significant difference of SBS between the base subgroups (B-AB/B-AB + SCA) and between the base + tooth subgroups (BT-AB/BT-AB + SCA) (*P* < 0.001), while no significant difference was detected between the tooth subgroups (T-AB/T-AB + SCA) (*P* < 0.903). Among all thermal-cycled groups, BT-AB + SCA showed the highest SBS (20.05 ± 0.49 MPa), while T-AB had the lowest SBS value detected in this study (14.85 ± 0.54 MPa).

Looking at the effect of thermal cycling on SBS, all groups showed significantly lower SBS values after thermal cycling (*P* ≤ 0.004) except the BT-AB group (*P*=0.096); however, the results were still significantly higher than the controls (*P* < 0.001). The results of all tested specimens before and after thermal cycling showed the same relation of alumina-blasting combined with SCA being better in SBS values than alumina-blasting alone for each treated surface (base/tooth/both) independently as shown in Table 2.

**Figure 2:**
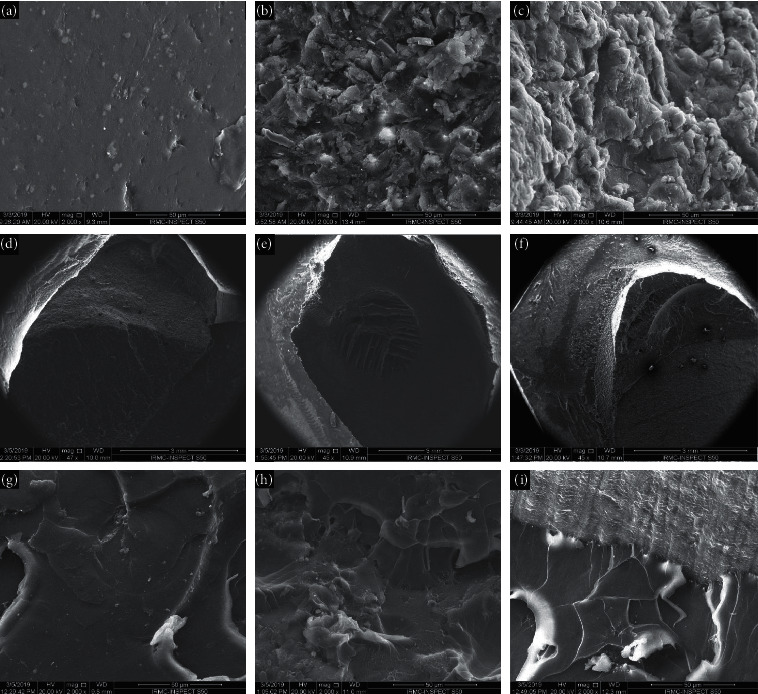
Representative SEM images showing (a) surface treatment effect on the control specimen before repair; (b) effect of AB surface treatment on the specimen before repair; (c) effect of AB + SCA surface treatment on the specimen before repair; (d) brittle fracture of the control specimen; (e) ductile fracture of the AB-treated specimen; (f) ductile fracture of the AB + SCA-treated specimen; (g) adhesive failure of the control specimen; (h) cohesive failure of the AB-treated specimen; (i) mixed failure of the AB + SCA-treated specimen.

**Figure 3:**
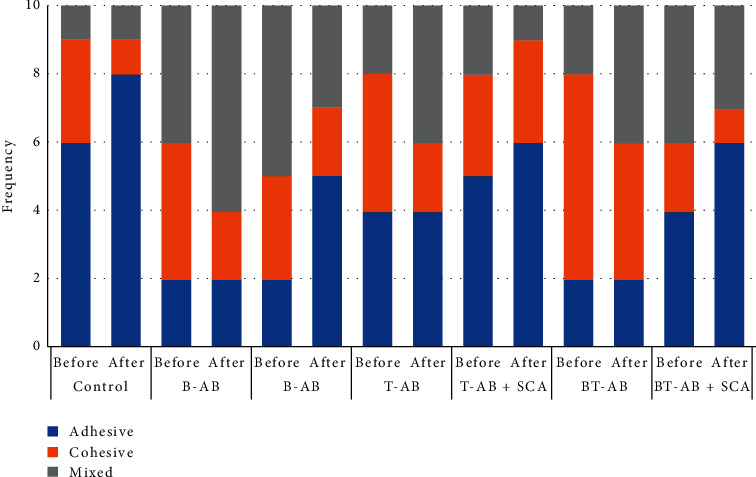
Summary of the nature of failure before and after thermal cycling.

The effect of surface treatment on teeth/bases and the fractured surface images under the SEM are shown in Figure 2. The control group showed smooth surfaces with little scratches (Figure 2(a)). The alumina-blasting caused the surface to be pitted and irregular (Figure 2(b)). SCA application resulted in less irregular surface than alumina-blasting with the surface topography showing shallow valleys and hollows (Figure 2(c)). Representative SEM images of the fractured surfaces showed smooth surface and few lamellae on a mirror-like appearance background for control specimens (Figure 2(d)) exhibiting brittle fracture characteristics. While in alumina-blasted and alumina-blasted with SCA groups, more changes in the surface topography were observed where multiple lamellae and depressions were evident (Figures 2(e) and 2(f)) exhibiting ductile fracture characteristics. As shown in SEM images for the nature of failure, three types of failures were observed: adhesive (Figure 2(g)), cohesive (Figure 2(h)), and mixed (Figure 2(i)). Control group and T-AB + SCA before/after thermal cycling, in addition to both B-AB + SCA and BT-AB + SCA groups after thermal cycling, failed mainly adhesively, while all the other groups generally showed mixed or cohesive failures (Figure 3). Mann–Whitney test results showed that thermal cycling had no significant effect on adhesive (*P*=0.291) and mixed failures (*P*=0.845), while a significant effect was detected for cohesive failures (*P*=0.042).

## 4. Discussion

SBS is the bond strength between two materials and how much they resist the load until they slide against each other and fracture or separate. SBS is one of the important mechanical properties that gives the PMMA its strength, and accordingly, it was tested in this study [18]. Oral cavity is subjected to variant temperatures [20] ranging between 4 and 60°C normally and usually simulated in the in vitro researches by the thermal cycling process [18]. Simply, thermal cycling represents mechanical fatigue in a moist oral condition [21]. This study was conducted to evaluate the effect of variant surface treatments and thermal cycling on SBS between repaired artificial acrylic teeth and PMMA denture base resins. The null hypothesis of neither surface treatments nor thermal cycling would affect the SBS was rejected.

According to the results of this study, it was found that alumina-blasting treatment increased the SBS as it was applied to any (base or tooth) or both surfaces. This increase could be attributed to the effect of abrasion achieved by aluminum oxide particles hitting the surface increasing its total surface energy and roughening the surface through formation of irregularities and undercuts, creating micromechanical retention pits for the repair resin and eventually increasing the bond strength [22]. In addition, alumina-blasting increased the bond strength by increasing the total bonding area which resulted in SBS improvement [17, 23]. In agreement with Meng et al. [9], the SBS between the repaired tooth and denture base was improved by treating the tooth ridge-lap surface with alumina abrasive particles.

The results showed that SCA application on the alumina-blasted teeth and/or bases significantly improved the SBS. This could be explained by the effect of the functional groups of SCA that enhanced the formation of covalent bonds between the PMMA, silane, and repair material [15]. In addition to this chemical bond, SCA with the help of its low viscosity [15] infiltrated the micropores on the surfaces facilitating the interlocking of the repair material within the irregularities formed by alumina-blasting. SCA was proved to enhance the repair bond by providing both chemical and micromechanical effects at the repair surface area [15]. The results of this study agreed with those of Lang et al. and Qaw et al. [10, 15] where the combination of mechanical surface treatment as alumina-blasting and chemical surface treatment as bonding agents significantly improved the repair bond strength.

According to the SEM examination, the improvement of the SBS of the alumina-blasted surfaces could be explained by the microretentive areas (Figure 2(b)) that increased the total adhesion area compared to the relatively smooth surface of the control group (Figure 2(a)). These microretentive areas were occupied by SCA which acted as an intermediate bonding agent between the repaired surface and the repair resin causing further increase in the SBS (Figure 2(c)). SEM analysis confirmed the improved SBS of treated groups, as the fracture mode was ductile in nature exhibited by the presence of irregularities, roughness, and a great number of lamellae on the fractured surface (Figures 2(e) and 2(f)). On the other hand, the brittle fracture was observed with the control group which showed a lower number of lamellae with smooth background indicating a weak bond (Figure 2(g)).

Comparing all the study groups before and after thermal cycling, the surface-treated groups showed significantly better SBS values than the controls even though there was a decline in the SBS after thermal cycling. The percentage of the decline was higher in the groups that were treated by combination of alumina-blasting and SCA in comparison with those that were treated by alumina-blasting alone (Table 2). The highest decline of SBS value after thermal cycling was 16.14% for BT-AB + SCA, followed by 15.61% for T-AB + SCA, while the decline was only 2.85% for BT-AB and 6.07% for T-AB. The decline in SBS after thermal cycling could be attributed to its effects on the interface between the two different materials where it causes degradation of the bond strength through repeated expansions and contractions, owing to temperature changes [18, 24]. Thermal cycling and immersion in water with different temperatures also cause degradation of the resin polymer itself [25, 26]. In addition, high water temperature promotes its rapid ingress into the resin mass [26]. The absorbed water and fluctuating temperatures could decrease the denture mechanical properties where the water fills the interpolymeric chain spaces moving them apart and making their slippage over each other under stress easier, which explains the low SBS [25, 27]. Marra et al. [16], in a study conducted in 2009 to determine the effect of thermal cycling on the SBS between the acrylic teeth and denture bases, concluded that thermal cycling caused significant reduction in the SBS, similar to the results seen in this study. Our results disagreed with those of Pande et al. [18] who found that the SBS between teeth and denture bases was not significantly affected by thermal cycling; however, their evaluations were not done for repaired teeth.

The control group failed mainly adhesively which suggests a weak repair bond, and the aim of the study was to improve this bond and have cohesive or mixed failures instead of the adhesive one. All the test groups before thermal cycling showed a less or equal number of adhesive failures against cohesive and mixed failures, which again supports our results of the increased SBS (Figure 3). Fortunately, after thermal cycling, the groups that were treated by alumina-blasting alone did not change in the number of adhesive failures, but the mixed failures increased, and the cohesive failures decreased significantly (*P*=0.042). On the other hand, all the groups treated with combination of alumina-blasting and SCA showed increased number of adhesive failures after thermal cycling, but this increase was not statically significant (*P*=0.291), and these groups had better SBS values than the controls, indicating that SCA might not have deteriorated during the process. The thermal cycling effect on the alumina-blasting and SCA is still not clear and needs further investigations.

The clinical importance of this study includes the new technique of teeth repair that can be used to decrease the chance of repeated teeth debonding and repair, hence saving the patient the cost of repair and dentist/patient time and improving the patient quality of life. The present study tested the repair bond of conventional acrylic resin. It would be interesting in the future to test recently introduced technologies, such as CAD/CAM dentures [28] or 3D-printed dental prostheses [29]. Additionally, further investigations are needed of more than 5,000 thermal cycles to evaluate the durability of SCA over extended period of use. Different properties can be also investigated such as tensile bond strength and impact strength. The limitations of this study include the study being done in vitro which does not completely resemble the oral environment. Many important clinical factors including denture shape and dimension, masticatory forces, and saliva moisturization were not included in this study which might affect the results. Further investigations are needed in vivo or/and in better clinical representation of the in vivo situation like further aging processes or more than 5,000 thermal cycles and different bonding agents.

## 5. Conclusions

The combined chemomechanical treatment using alumina-blasting and silane coupling agent for the denture base and teeth improves the shear bond strength of the repaired tooth/denture base. Thermal cycling decreases the shear bond strength of all groups, particularly the ones with combined chemical and mechanical surface treatment.

## Figures and Tables

**Table 1:** Specimen grouping and specifications.

Groups according to treated side	Code	Treatment specifications
No treatment	Control	Untreated heat-polymerized denture base material

Base (B)	B-AB^(1)^	Denture base material treated with alumina airborne abrasion
	B-AB + SCA^(2)^	Denture base material treated with alumina airborne abrasion and SCA

Teeth (T)	T-AB	Teeth treated with alumina airborne abrasion
	T-AB + SCA	Teeth treated with alumina airborne abrasion and SCA

Base + teeth (BT)	BT-AB	Denture base material and teeth treated with alumina airborne abrasion
	BT-AB + SCA	Denture base material and teeth treated with alumina airborne abrasion and SCA

^(1)^AB: alumina-blasting and ^(2)^SCA: silane coupling agent.

**Table 2:** Mean shear bond strength ± SD (MPa) and significance of repaired specimens with different surface treatments at 0 and 5,000 thermal cycles.

Treated side	Group	0 thermal cycles, mean ± SD	5,000 thermal cycles, mean ± SD
No treatment	Control	9.78 ± 0.81	6.97 ± 0.50

Base	B-AB	17.02 ± 1.00^a^	15.74 ± 0.72^a^
	B-AB + SCA	21.59 ± 1.10	18.61 ± 0.76^b^

Tooth	T-AB	15.81 ± 0.60^a^	14.85 ± 0.54^c^
	T-AB + SCA	18.00 ± 1.10^a,b^	15.19 ± 0.71^a,c^

Base and tooth	BT-AB	18.98 ± 0.60^b,A^	18.44 ± 0.76^b,A^
	BT-AB + SCA	23.91 ± 0.96	20.05 ± 0.49

Groups with similar letters are not significantly different from each other. Vertically identical superscripted small letters denote no significant differences among groups (*P* > 0.05). Horizontally identical superscripted capital letters denote no significant difference after thermal cycling within the surface treatment group (*P* > 0.05).

## Data Availability

The data used to support this study are available upon request from the corresponding author.
